# “Molecular Anatomy”: a new multi-dimensional hierarchical scaffold analysis tool

**DOI:** 10.1186/s13321-021-00526-y

**Published:** 2021-07-23

**Authors:** Candida Manelfi, Marica Gemei, Carmine Talarico, Carmen Cerchia, Anna Fava, Filippo Lunghini, Andrea Rosario Beccari

**Affiliations:** 1 Dompé Farmaceutici SpA, Via Campo di Pile, 67100 L’Aquila, Italy; 2 Department of Pharmacy, University of Naples “Federico II”, 80131 Napoli, Italy

**Keywords:** Scaffold analysis, HTS data analysis, Library design, Network visualization, Clustering

## Abstract

The scaffold representation is widely employed to classify bioactive compounds on the basis of common core structures or correlate compound classes with specific biological activities. In this paper, we present a novel approach called “Molecular Anatomy” as a flexible and unbiased molecular scaffold-based metrics to cluster large set of compounds. We introduce a set of nine molecular representations at different abstraction levels, combined with fragmentation rules, to define a multi-dimensional network of hierarchically interconnected molecular frameworks. We demonstrate that the introduction of a flexible scaffold definition and multiple pruning rules is an effective method to identify relevant chemical moieties. This approach allows to cluster together active molecules belonging to different molecular classes, capturing most of the structure activity information, in particular when libraries containing a huge number of singletons are analyzed. We also propose a procedure to derive a network visualization that allows a full graphical representation of compounds dataset, permitting an efficient navigation in the scaffold’s space and significantly contributing to perform high quality SAR analysis. The protocol is freely available as a web interface at https://ma.exscalate.eu.

**Supplementary Information:**

The online version contains supplementary material available at 10.1186/s13321-021-00526-y.

## Introduction

High-throughput screening (HTS) of small-molecule libraries is routinely used in drug discovery process to identify novel leads against clinically relevant targets. Successful HTS require high quality, validated screening assays, but also an effective strategy for chemical structures selection is fundamental for the following hit-to-lead phase. HTS libraries, indeed, comprise some hits of interest, but also many compounds resulting in false positives or promiscuous hits, as well as number of compounds with no relevant biological activities at micromolar concentrations in biochemical assays [1]. The first fundamental step, affecting the probability of success of the entire lead discovery process, is represented by an incisive preliminary structure activity relationships (SAR) analysis. One of the crucial tasks in the design of large diverse libraries is the chemical space mapping. Selection of a representative subset of the desired chemical space is generally addressed by the identification of three elements: a set of meaningful descriptors [2], a similarity metric allowing to compare molecular structures pairwise [3], and a clustering algorithm for grouping structures according to the calculated pairwise similarity values [4, 5]. Many clustering algorithms exist [6], and many clustering techniques are able to address this task for groups of 10^5^ to 10^7^ compounds; however, the identification of relevant chemical series within the generated clusters is much more difficult. The generation of clusters organized as “series” in medicinal chemistry is an important asset of the scaffold-based techniques.

A chemical scaffold, also referred to as ‘chemotype’ or ‘Markush structure’, can be defined as the common structure characterizing a group of molecules. Compounds sharing the same scaffold are likely to have a similar synthetic pathway as resulting from the concept of molecular template in combinatorial chemistry [7]. Once a scaffold is defined, SAR can be developed analyzing the effects of the substitution patterns [8]. The scaffold approach shows several advantages, in particular its outcomes are both simple to interpret and medicinal chemistry-oriented; additionally, some of the most significant features of the graph-based approaches [9] are combined with molecular fingerprint characteristics and maximum common substructure methods. Bemis et al*.* [10] introduced a systematic analysis of drugs according to their scaffold/framework representation which it is now a well-established method alongside molecular descriptors, molecular fingerprints and graphs. In the last twenty years, different scaffold definitions have been introduced and numerous scaffold-based computational approaches have been developed for structural classification and biological activities prediction [11]. The introduction in 2005, by Wilkens et al. [12], of hierarchies based on several kinds of scaffold deconstructions, represented a milestone in the development of scaffold-based approaches. In 2007, Schuffenhauer et al. [13] demonstrated the potentiality of combining the scaffold-based approach with ad hoc graphical representations through the “Scaffold Tree” algorithm and visualization tool; Schuffenhauer also introduced a rule-based ring disassembly. Since then, other decomposition and visualization tools have been developed, such as Scaffold Hunter in 2009 [14], recently revised and extended, and Scaffold Explorer in 2010 [15]. In 2008 Gianti and Sartori [16] proposed an alternative procedure to address scaffold decoration, pruning and fragmentation as a workflow for the identification of “privileged fragments”. Agrafiotis et al. [15], in 2010, demonstrated that the inclusion of relevant side chains and functional groups in the scaffold representation could greatly enhance the derivation of robust SAR, thus indicating that the explicit consideration of the most significant molecular features overcomes the limits associated with “a priori” definition of specific pruning rules. In 2011, Varin et al. [17] proposed an extended version of the previously developed Scaffold Tree and demonstrated that rule-based approaches in fragmentation are less useful and flexible than the unbiased ones. Lipkus [18] introduced hierarchies between different abstraction levels and, finally, different hierarchical scaffold decomposition and abstraction approaches were proposed by many authors [19, 20].

All the above described methods share two major limitations; first a single ring system is represented, decorated with chains of various length, therefore, when pruned, all the molecules collapse into a degenerated cluster. Additionally, there is no relationship between scaffolds when the difference is represented by one or more ring systems. These issues are particularly limiting for the analysis and selection of vendor libraries to build diverse compound collections and, afterward, for HTS campaigns analysis in order to obtain preliminary SAR.

Very recently, Bandyopadhyay et al. [21], in order to overcome the limits related to hard clustering methods, which assign each molecule to a single cluster and so tend to place structurally analogous molecules into different and not related clusters, described a method based on fuzzy clustering that may assign a molecule to different clusters. In this method, for each molecule an exhaustive enumeration of Bemis-Murcko scaffolds, corresponding to all possible combinations of ring systems, was applied and data were annotated and aggregate at scaffold level, allowing to relate molecules on the basis of shared scaffolds. Another recent approach to perform scaffold analysis is based on retrosynthesis rules, which allow to easily identify analog series [22, 23]. Such analog series-based scaffolds can also be associated with activity information to develop possible target hypotheses for other compounds containing the same scaffold [24]. The organization of compounds in analog series leads to the formation of “constellations” of molecules, in chemical space, which can be visualized as a network of all possible molecule–core relationship [25].

However, the main limitation of the network connecting molecules and scaffolds generated with these implementations is that they are based on a unique scaffold representation, not sufficient to effectively map the chemical space of a heterogeneous ensemble of molecules, for example multi-scaffold libraries, and to capture relationships with the relative biological activity. The critical point is that it is not possible to define a priori the best representation of a molecule, because it mainly depends on the biological context and on the nearest-neighbors of the screened library.

Here, we present a novel approach, called “Molecular Anatomy”, for the generation and analysis of correlated molecular frameworks aimed at overcoming the limitations of scaffold analysis based on a single predefined set of rules. In our experience, the here identified molecular frameworks and related fragments are able to capture most of the structure activity information from HTS campaigns, and are also useful for other applications, such as library design and analysis. In particular, the combination of fragments, correlated in frameworks and wireframes, identifies relevant chemical moieties in an efficient manner, clustering together many scaffolds with similar shapes despite, for example, different dispositions of heteroatoms or small differences in bond order. To the best of our knowledge, this is a distinctive feature of our approach, compared to other known methodologies, such as the widely accepted Maximum Common Substructure (MCS) [26].

In the “Methods” section, the molecular scaffold representations and the fragmentation rules used to generate the related fragments are defined. A COX-2 inhibitors dataset has been chosen to illustrate our approach. Then, we introduce an innovative network representation as a more convenient tool for SAR evaluation and visualization. We first apply this visualization to the molecular frameworks proposed, and then we extend the network visualization also to the underlying fragments, to show the full graphical representation. We also summarize the main advantages of our method compared to the other approaches proposed so far. Finally, we show the general applicability of our approach by performing the SAR analysis of 26,092 commercial compounds tested in an HTS campaign aimed at identifying potential inhibitors of the enzyme Histone deacetylase 7 (HDAC7).

## Methods

### Dataset definition

*COX-2* A dataset containing COX-2 inhibitors was prepared and used to illustrate the “Molecular Anatomy” approach. To this aim, the Integrity™ database was interrogated to search for COX-2 inhibitors, providing 2599 molecules in total. Of these, 816 were in preclinical phase or in a higher phase of clinical development. This subset was used in the following analysis to compare different scaffold representations. A Pipeline Pilot protocol [27] was used to standardize the molecular structures, to classify them according to the molecular mechanism (e.g. inhibitors) and highest phase of development, to perform substructure searches, to generate molecular frameworks according to our definition rules and, finally, to analyze the results in order to compare the different scaffold definitions.

*HDAC7* dataset of 26,092 commercial compounds, tested as potential HDAC7 inhibitors during an HTS campaign performed internally within Dompé, was used as a more complex case study. The compounds were stratified in different activity classes according to the value of percent inhibition of the HDAC7 activity obtained at 10 μM concentration (Table 1).

**Table 1:** Activity thresholds for 26,092 commercial compounds tested at 10 μM concentration against HDAC7, classified on the basis of enzyme activity percent of inhibition

Activity class	N. of compounds	% of inhibition
Inactive	23,750	< 19
Weak	2141	19–33
Moderate	144	33–50
Strong	37	50–80
Very strong	20	> 80

### ChEMBL datasets

Additional compound datasets were retrieved from release 28 of ChEMBL [28, 29] for further proof-of-concept studies. Two sets were selected, with at least 1000 active compounds, identified with ChEMBL Target IDs 202 (Dihydrofolate reductase) and 2000 (Plasma kallikrein). Only data measuring binding of compounds (i.e., assay type “B”) were collected. To ensure a high level of data integrity, only compounds with explicitly defined IC_50_ values were selected, using a cut-off of 5 μM as minimum potency to define compounds as “actives”. A third dataset consisted of a recently added repository generated within the “EXaSCale smArt pLatform Against paThogEns for Corona-Virus, Exscalate4CoV or E4C” project (CHEMBL4495564), containing activity data for ~ 8000 compounds from the primary screening against SARS-CoV-2 Main protease (Mpro) [30]. In this case, compounds were considered as actives if enzyme inhibition was at least 40%.

### Identification of common scaffold representations and evaluation of their performance

**Fig. 1:**
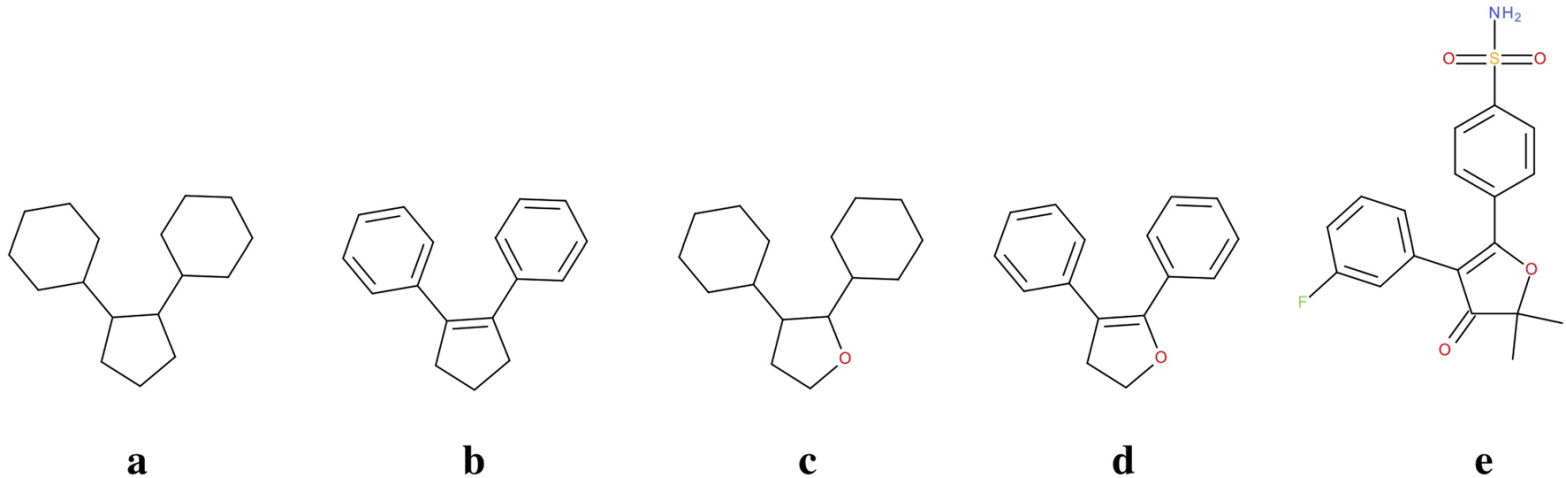
Possible scaffold definitions for the COX-2 inhibitor Polmacoxib: **a** is the most abstracted representation, obtained removing both bond and atom type; **b** and **c** representations retain, respectively, only bond and atom type; **d** corresponds to the Bemis-Murcko scaffold; **e** is the full structure of the Polmacoxib

In theory, it is possible to define an arbitrary number of scaffold's representations based on different levels of abstraction and pruning rules. Figure 1 shows an example applied to the COX-2 inhibitor Polmacoxib (whose full structure is depicted in panel e) [31].

Panel 1a shows the most abstracted representation (hereafter 1a), obtained removing both bond and atom type. This representation is also known as “cyclic skeleton”. Representations 1b and 1c retain only bond and atom type, respectively, whereas the 1d representation corresponds to the Bemis-Murcko scaffold, containing all the rings and chains connecting them of the original molecule.

**Fig. 2:**
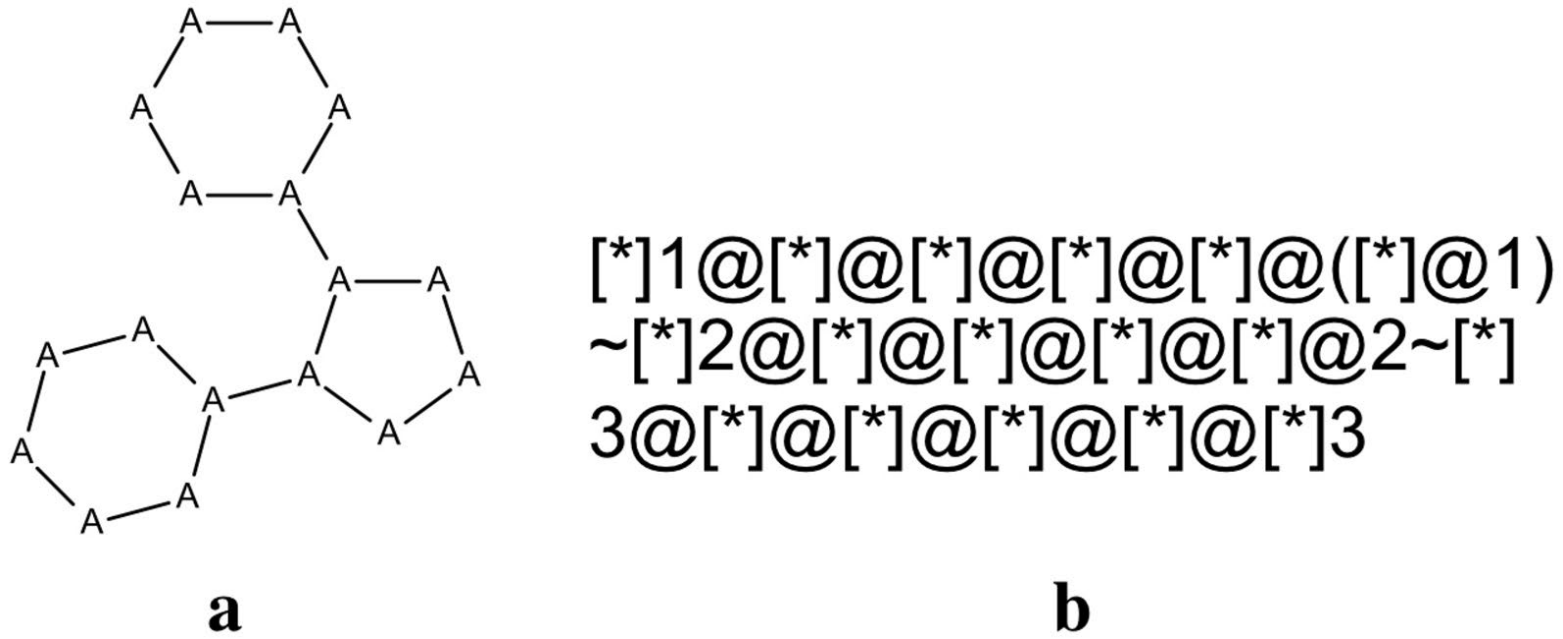
**a** MDL substructure query of the COX-2 inhibitors most common moiety (any atoms and any bonds and **b** the corresponding SMARTS string

By using the most abstracted representation 1a of the Polmacoxib scaffold, corresponding to the most common moiety of COX-2 inhibitors, a subset of 224 COX-2 inhibitors was identified. Figure 2 reports the MDL substructure query and the corresponding SMARTS string used to retrieve the molecules containing this substructure.

These 224 molecules correspond to 84 different scaffolds if the less abstracted 1d representation is used (Table 2), thus resulting impossible to associate them each other as belonging to the same substructure. Furthermore, we found that 53 out of 84 Bemis-Murko scaffolds (63.1%), have one or more additional rings, corresponding to 82 of the 224 molecules (36.6%) and clustered in 34 groups according to the 1a representation, whereas the remaining 142 molecules, with exactly 3 rings, corresponding to 31 scaffolds based on the 1d representation, collapse to only one cluster if the 1a representation is used.

**Table 2:** Clusterization of the 816 COX-2 inhibitors in preclinical development or in a higher phase, and of the subset of those matching the MDL substructure reported in Fig. 2. Clusters were obtained for each representation type, distinguishing the number of those containing only molecules with exactly 3 or more than 3 rings

	All	Matching the MDL substructure	With only 3 rings	With more than 3 rings
N. of molecules	816	224	142	82
Representation type	N. of clusters			
1a	277	35	1	34
1b	366	59	11	48
1c	412	78	27	51
1d	434	84	31	53

### Identification of nine correlated scaffold representations

**Fig. 3:**
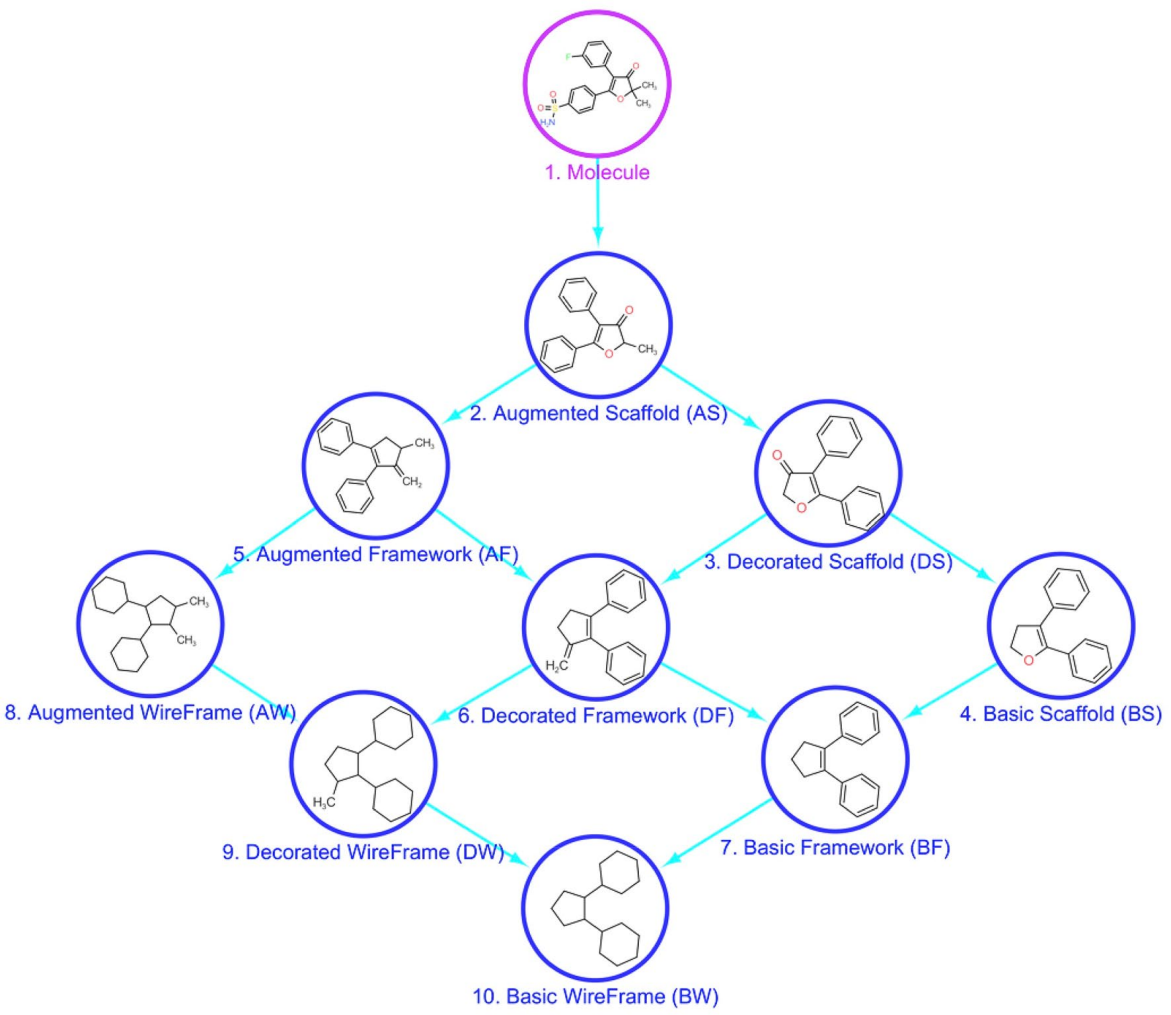
Representation of the nine levels of structure decomposition for the COX-2 inhibitor Polmacoxib

In “Molecular Anatomy” we used, as starting point, the widely accepted scaffold abstraction representation (here called *Basic Scaffold*), which is generated by removing all side chains and terminal atoms. Then, we defined a set of nine molecular frameworks (MF) at different abstraction levels to match different side chain definitions, as showed in Fig. 3 for the COX-2 inhibitor Polmacoxib. We used two sets of pruning rules able to determine a multidimensional hierarchy.

**Fig. 4:**
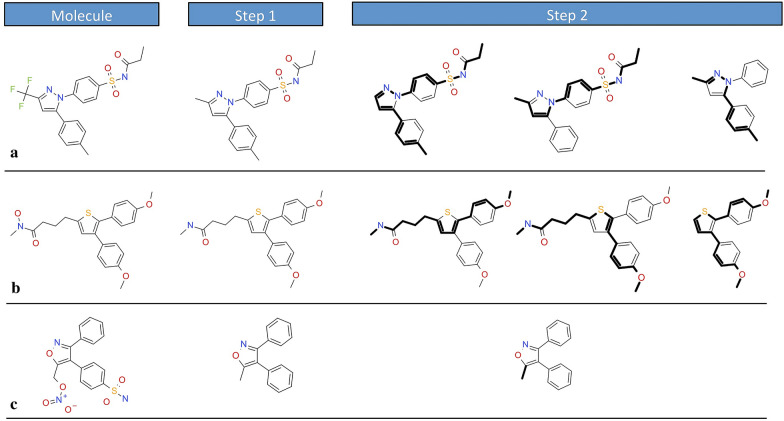
Augmented scaffold rules to identify the longest atom path: in the first step, all terminal non carbon atoms with a bond order equal to 1 are removed, in the second step the longest carbon chain ending with a carbon atom is identified. Three examples explain some possible cases (**a**) three different paths can be identified and the longest is retained (the first); **b** three paths can be identified two of them are the longest with the same length and the first identified is retained; **c** only one path can be identified

The first set of rules is based on an increased level of structural information with respect to the basic scaffold. In a first step, terminal atoms with bond order greater than one are maintained (*Decorated Scaffold*); in a second step, the longest atom chain, considering also substitutions, is retained but all terminal non-carbon atoms, belonging to side chains, are iteratively pruned (*Augmented Scaffold*). In case that no terminal atoms remain removing all terminal non-carbon atom with a bond order equal to 1, decorated and augmented scaffold coincide. Some examples reported in Fig. 4 explain these rules, applied to different possible cases.

The second set of rules, conversely, increases chemical abstraction by removing the atom type label and then the bond order, generating, respectively, a *Framework* and a *Wireframe* for each level of the scaffold (basic, decorated and augmented), thus finally producing nine molecular representations with a hierarchical correlation.

### Fragmentation rules definition

**Fig. 5:**
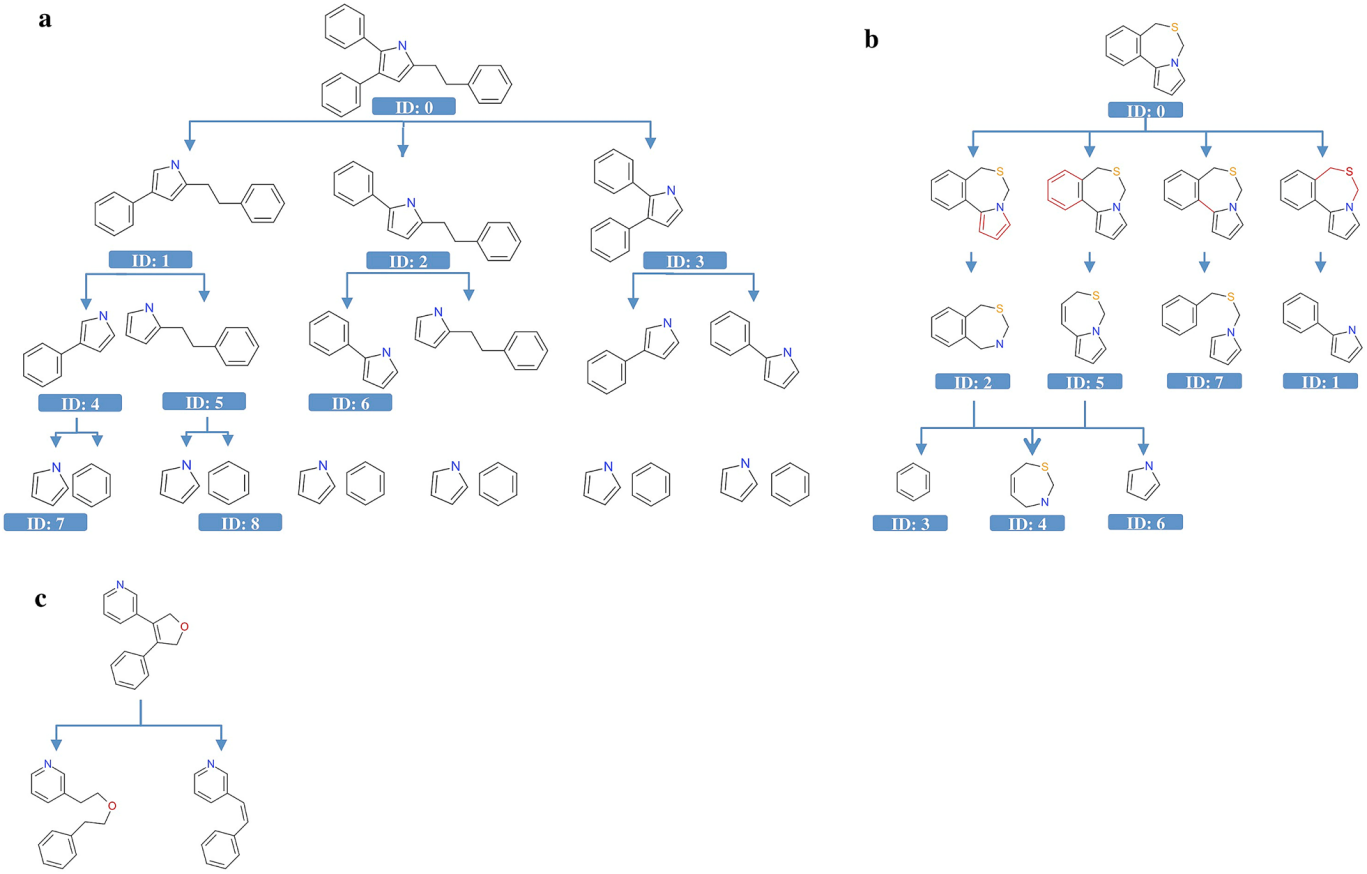
Representation of the hierarchical decomposition of a molecule by the exhaustive and progressive elimination of (**a**) all the chains from the scaffold, **b** all fused rings from the scaffold, and **c** all internal rings from the scaffold

To further overcome one general limitation of the scaffold based techniques [12, 14, 32] that, by definition, molecules sharing the same scaffold only partially belong to distinct clusters, in “Molecular Anatomy” approach we have implemented an unbiased fragmentation scheme that can be applied in parallel to all nine scaffold representations described above. These rules are explained in Fig. 5, applied to specific molecules chosen on representative purpose. The first rule (Fig. 5a) depicts an example of fragmentation based on an exhaustive and progressive elimination of all the internal chains from the scaffold. As second rule, unbiased ring disassembly was also implemented; the methodology for ring decomposition involves the removal of all fused rings, allowing their opening into fragments (Fig. 5b). For sake of consistency, we also introduced a third rule to remove internal rings (Fig. 5c).

**Fig. 6:**
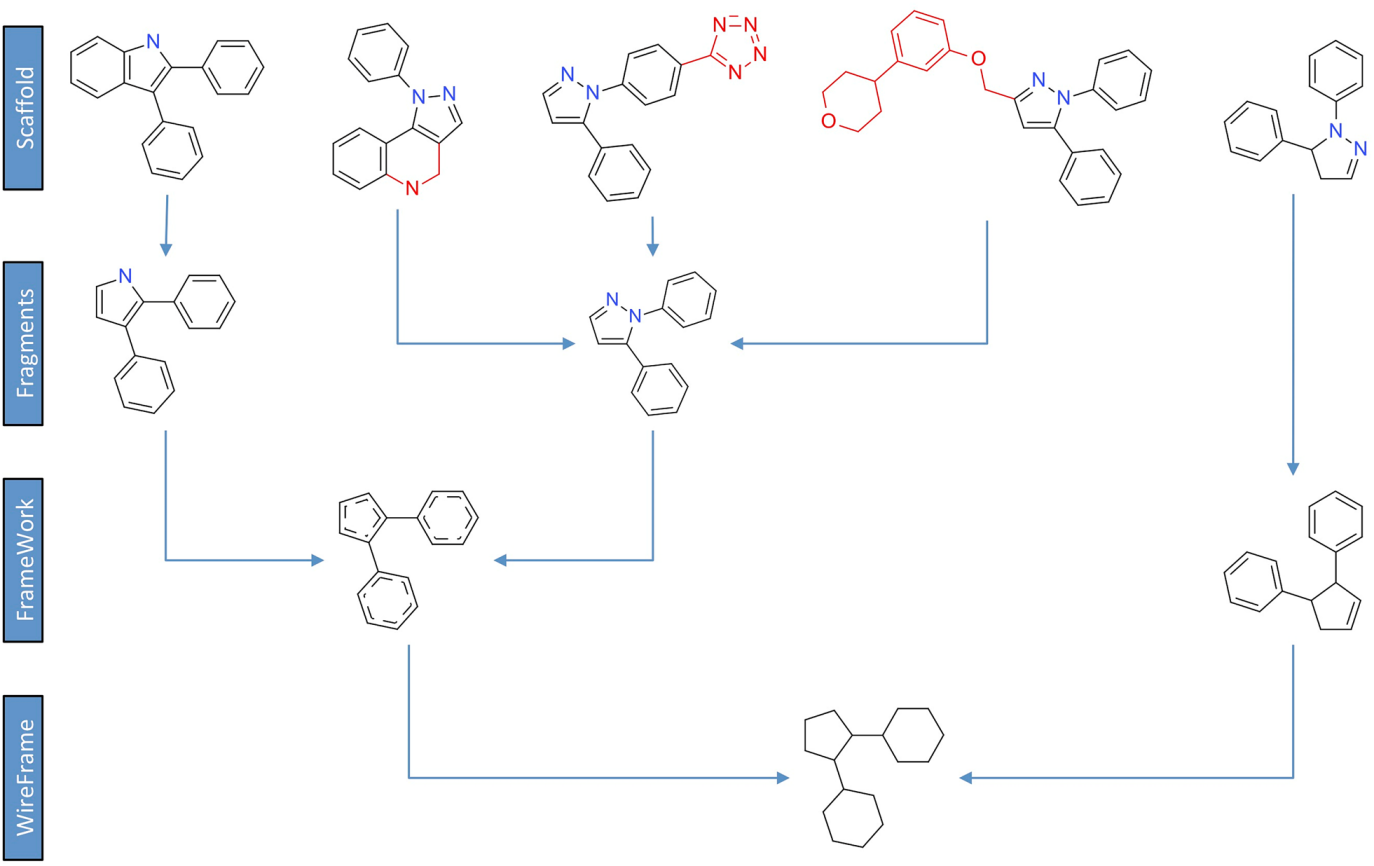
Fragments and molecular frameworks at different abstraction levels can be combined to overcome the limits of an approach based on a single representation

The here reported fragmentation and deconstruction introduce other hierarchies, meaning that each fragment of the original scaffold is related with all the other representations in a multi-dimensional space. As a result of this multi-dimensional hierarchical scaffold analysis, the entire set of generated molecular frameworks are highly interconnected, and it is possible to move from one to another following SAR. To clarify this concept, Fig. 6 reports an example showing how the combination of fragments and molecular frameworks at different abstraction levels allows to cluster molecules with different scaffolds.

### Network representation of “Molecular Anatomy”’s frameworks

The software Cytoscape was used for creating and visualizing an MF-based network, which was also integrated with activity data for the SAR analysis. This network provides a full graphical representation of the dataset composition, allowing to easily navigate through the molecular frameworks and their hierarchical correlation. A Pipeline Pilot protocol was implemented to prepare the data matrix needed for the visualization, in the format required for the import process.

Each molecule from the dataset of 816 COX-2 inhibitors was described according to the nine molecular representations implemented in the “Molecular Anatomy” approach (Additional file 3: Table S2); then, a unique list of frameworks was obtained (Additional file 4: Table S3), keeping the less abstracted one in case of duplicate structures (when a same scaffold structure was obtained with different representations), corresponding to the nodes of the network. All possible parent–child relationships between the nine molecular frameworks of each molecule were generated, as reported in Additional file 5: Table S4, according to the hierarchical relationships between the representation types shown in Fig. 3, corresponding to the edges of the network.

To fully exploit this graphical representation, the network data matrix can be integrated with the enrichment factors (EF) calculated, according to Eq. (1), for each molecular framework (MF) according to the activity data of the corresponding molecules, keeping the highest EF value in case of duplicate structures when the unique list of frameworks is generated.

1 $${\text{E.F}} = \frac{{MF \ ratio}}{{Total \ ratio}}$$

where

2 $${\text{MF ratio}} = \frac{{MF \ actives}}{{MF \ actives + MF \ inactives}}$$

3 $${\text{MF actives}} = {\text{number of active molecules having a specific MF}}$$

4 $${\text{Total ratio}} = \frac{{Total~actives}}{{Total~actives + Total~inactives}}$$

In order to focus the network visualization on the most relevant dataset information, the nodes associated with EF = 0 can be filtered out, thus stepping through the nodes with increasingly higher EF values, as described in the HDAC7 case study (see “Results and discussion”).

### A fully connected network representation by means of “Molecular Anatomy”’s frameworks and fragments

“Molecular Anatomy” allows, as already described, to derive trees in multiple dimensions such as *wireframe* > *framework* > *scaffold*, or *augmented* > *decorated* > *basic* or *wireframe* > *ring disassembly* > *fragments* and in all the other possible directions maximizing the SAR information of the dataset. The network visualization can be extended also to the fragments to obtain a fully connected network, considering that the smallest fragments (e.g. benzene ring) are shared by a huge number of the original molecules. In this implementation, the network nodes can be molecular frameworks, fragments or entire small molecules, and the direction of the edges, defined by the fragmentation rule, starts from the originator fragment and end up into the corresponding fragments.

### Molecular Anatomy Web interface implementation

The above-described protocol is freely accessible at https://ma.exscalate.eu. The web interface was implemented using LAMP (Linux Apache MariaDB PHP), an open source Web development platform that allows a fluent and responsive user experience in displaying and handling the output data, which in this case are calculated on the fly in a completely automated Pipeline Pilot workflow.

### Implementation of Molecular Anatomy approach in Knime

The Pipeline Pilot protocol for the preparation of the data matrix was also re-implemented in Knime 4.3.2 by means of in-house Python scripts and taking advantage of the available RDKit [33] and Indigo nodes [34].

## Results and discussion

### Comparison between common scaffold representations and “Molecular Anatomy” to perform SAR analysis

As shown in “Methods” section for to the COX-2 inhibitors dataset, scaffold representations with high level of abstraction, showed in Fig. 1a–b for Polmacoxib, perform generally better than the others in the identification of relevant chemotypes. Table 2 summarizes the results obtained for each representation in terms of number of clusters generated, starting, on one hand, from all the 816 COX-2 inhibitors in preclinical development or in a higher phase, and, on the other hand, from the subset of the COX-2 inhibitors matching the MDL substructure reported in Fig. 2, the most common COX-2 inhibitor moiety. In particular, the number of clusters containing the molecules matching the common substructure with exactly or more than 3 rings was specified.

Representation 1a clusters together most of the well-known marketed drugs, such as valdecoxib and celecoxib, as well as many others leads and experimental drugs, and collapses all the 142 active molecules with exactly 3 rings to a single cluster. This cluster likely includes also several inactive molecules. Interestingly, we can note that, even though this representation is used, still almost the 40% of the scaffold information, corresponding to the molecules with additional rings, would be lost in unrelated clusters, impairing the identification of the most relevant additional structural information.

Using the less abstracted representation 1d, we can retrieve and distinguish the most diverse COX-2 inhibitor scaffolds, even if this information is distributed in 84 clusters considering both those with 3 or more rings. Furthermore, an intermediate representation as 1b, where only the atom type information is removed, could allow a more effective clustering of the relevant structural information, identifying only 11 different frameworks containing molecules with exactly 3 rings, instead of 31; but, almost the same number of clusters containing molecules with more than 3 rings is generated with the two representations (48 instead of 53).

This example on COX-2 inhibitors clearly shows how this kind of analysis strongly depends on the nature of the dataset; each scaffold abstraction of Fig. 1 provides some important structural information but none of them is sufficient, alone, to capture the complexity of the heterogeneous ensemble of molecules. Only the integration of the information captured from the different scaffold abstractions, in a Multi-Dimensional Hierarchical Scaffold Analysis, allows to effectively map the entire chemical space of multi scaffold libraries. Furthermore, the combination of the “Molecular Anatomy” approach, the fragmentation rules and the network representation allows to immediately focus the attention on the most interesting and useful structural information, easily navigating among several structural clusters, moving from a molecular framework to another on the basis of their hierarchy and according to the SAR.

**Fig. 7:**
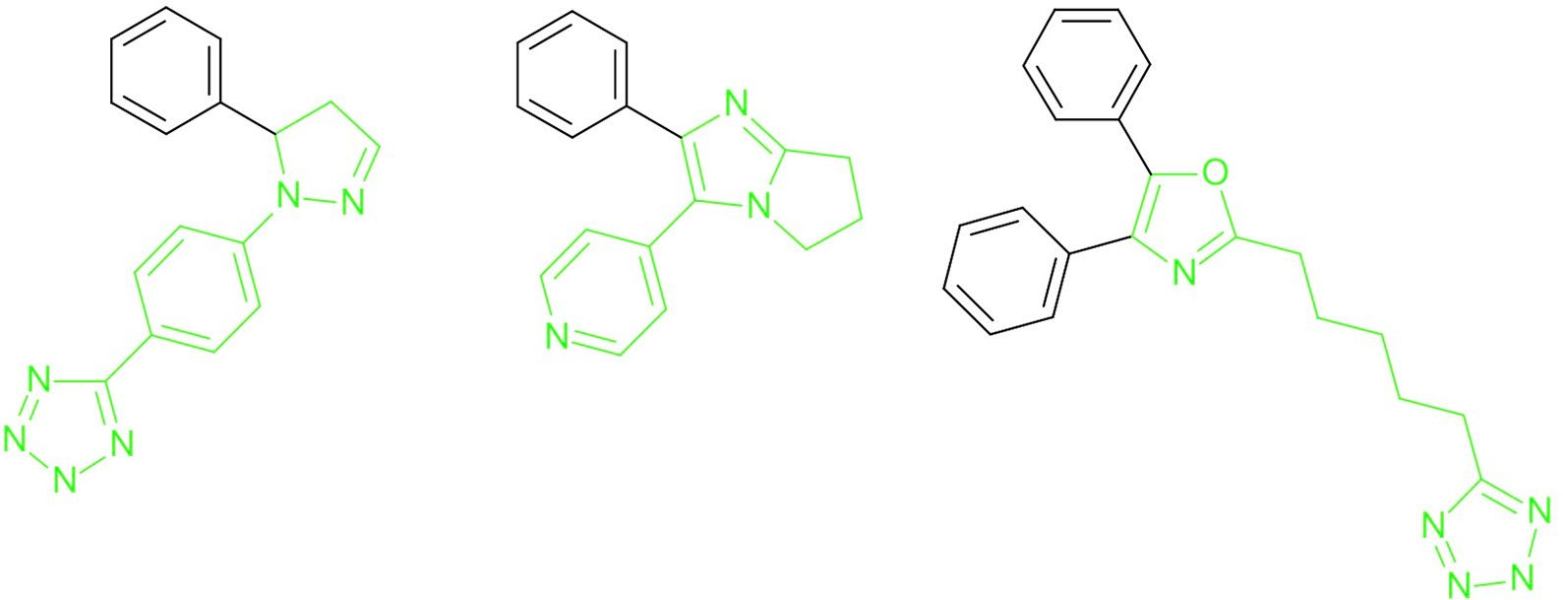
A tentative rule-based decomposition is represented in green; in this case the reasonable hypothesis that phenyl rings are less relevant than heterocyclic ones leads to sub-scaffolds lacking the COX-2 inhibitors moiety

Attempts to identify more relevant chemical moieties have been presented in the past, for example the rule-based decompositions proposed by Schuffenhauer et al. [32], schematized in Fig. 7 for three COX-2 inhibitor scaffolds. However, a clear limitation resides in the difficulty to define a priori a set of rules able to maintain a general consistency with SAR information.

The method that we propose, involving the combination of correlated molecular frameworks and fragments, is able to efficiently identify relevant chemical moieties, and to cluster together active molecules (also in the nanomolar range) belonging to different molecular classes within HTS campaigns, capturing most of the SAR information.

To fully exploit the hierarchical correlation among the molecular frameworks and to generate a full graphical representation of the analyzed dataset, we also propose a network visualization. Actually, the combination of the MF approach with a network representation provides a more convenient tool for SAR evaluation and visualization [35–38], usefully guiding the user from a molecular framework to another, on the basis of their hierarchy in the direction of increasing or decreasing level of abstraction and according to the SAR.

**Fig. 8:**
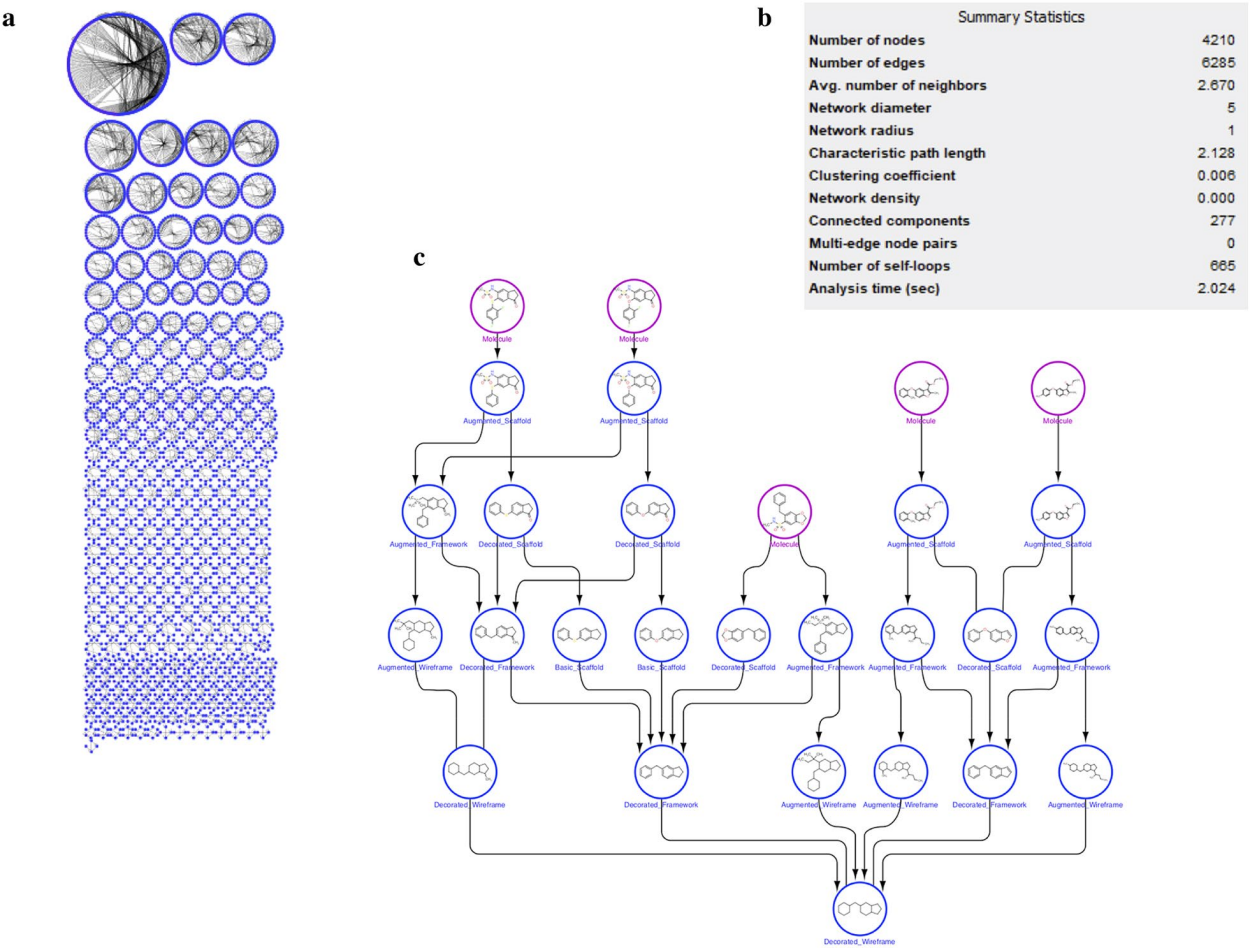
**a** Cytoscape network visualization of the 816 COX-2 inhibitors subset in preclinical development or in a higher phase; **b** main statistical parameters of the network; **c** hierarchical visualization of a smaller cluster used to show and explain the oriented network, where nodes are molecular frameworks connected in the direction of increasing abstraction level of the molecular representations

Figure 8 shows the complete network obtained for the dataset of 816 COX-2 inhibitors. As reported in the list of statistical parameters (Fig. 8b), 277 connected components were generated, corresponding to the clusters obtained using the most abstracted (basic wireframe) representation. It is possible to clearly note the biggest cluster at the top of Fig. 8a corresponding to the 142 molecules with exactly 3 rings (Table 2), all sharing the basic wireframe 1a. Figure 8c reports the hierarchical visualization of a smaller cluster, to further show how this graphical representation of the data matrix consists in an oriented network, where nodes are in general molecular frameworks, and the direction of the edges is defined by the direction of increasing abstraction level of the molecular representations.

**Fig. 9:**
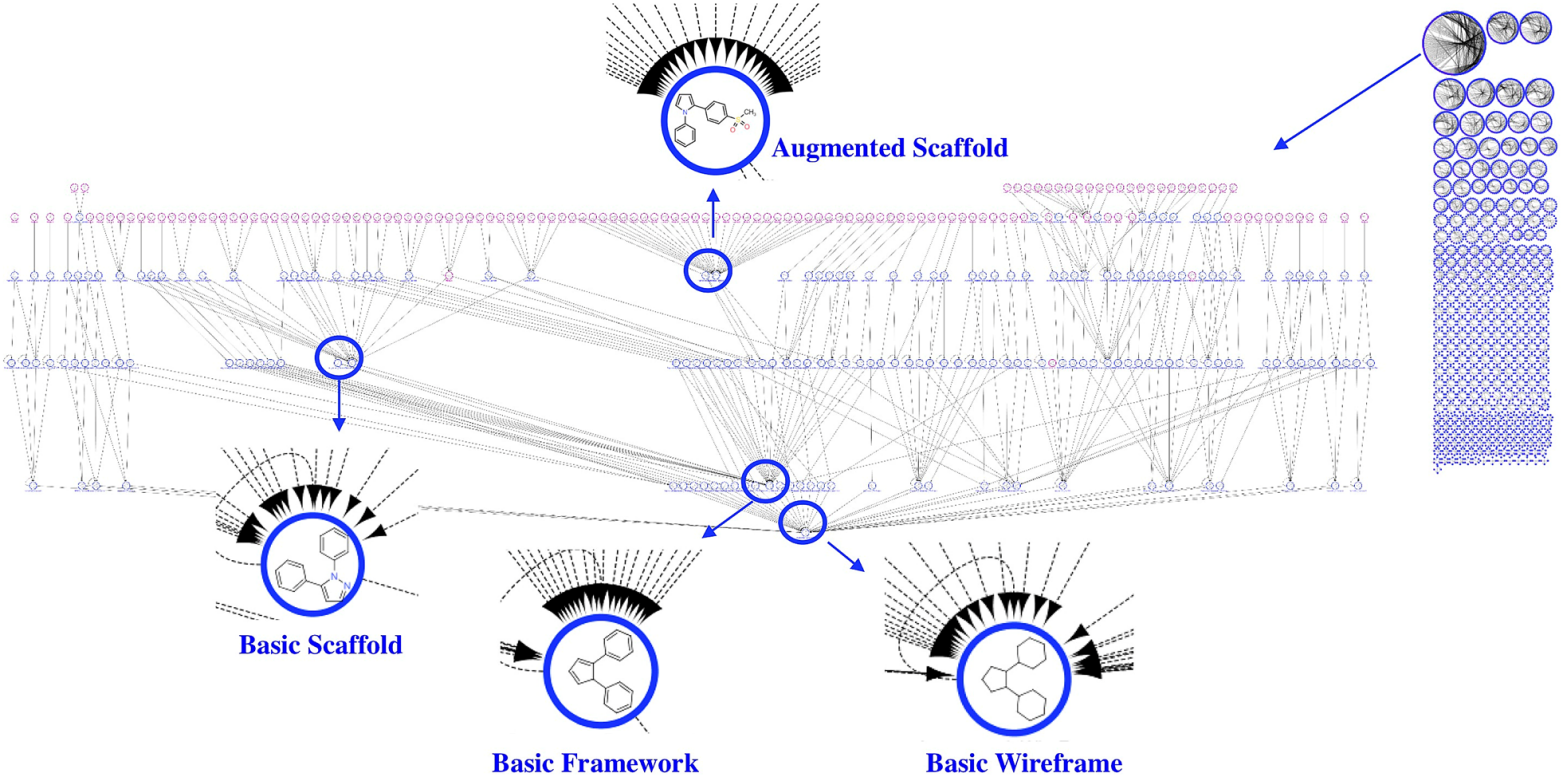
Visualization of the most populated cluster, starting from the network visualization of the 816 COX-2 inhibitors shown in Fig. 8a, corresponding to the 142 COX-2 inhibitors having the same basic wireframe, zoomed in the Figure. It is also possible to identify the molecular frameworks with more connection in the network and more representative of the active molecules (violet bordered)

Furthermore, it is possible to retrieve the relationships among the diverse representations within this cluster and, focusing on the most interconnected frameworks, to identify the structural characteristic representative of the active molecules, as shown in Fig. 9. On the other hand, the network visualization clearly shows the high number of singletons that would be dispersed considering only the representation 1a. Here, thanks to the use of the fragmentation, these singletons can be related each other if containing the same fragments, allowing to easily verify if they contain characteristics in common with relevant clusters of actives.

Focusing on the fragments related to the basic wireframe representation, all the clusters identified in Fig. 8a can be connected each other in a unique network, as can be visualized in Additional file 1: Figure S1.

Furthermore, Additional file 1: Figure S2 shows the fragments with the highest indegree value, in particular cyclohexane and cyclopentane, which means the highest number of fragments connected within the network in Additional file 1: Figure S1.

Some qualitative considerations about the obtained networks can be done. As a first point, it is reasonable that highly connected singletons tend to be small fragments shared by a large number of molecules included in the library (as shown in Additional file 1: Figure S2). On the contrary, low molecular weight singletons involved in a small number of connections represent potential interesting decorations of a specific group of the original molecules. If this group is enriched in a specific activity of interest, the corresponding singleton fragments connecting all the molecules included in the group, could represent a pharmacophore. As a second point, high molecular weight singleton fragments, connecting cluster of molecules with enriched activity, could represent chemical scaffolds or the “minimal chemical entity” that confers the selected activity to the cluster. As a third point, it is comprehensible that the meaning of the singleton constituting the networks may change according to the fragmentation rules used. While the approach suggested herein consists in a purely informatics fragmentation procedure, an alternative method is possible, where singletons consist in reaction intermediates derived applying retrosynthetic rules to the original molecules. In other words, in this case the network would contains, as “fragments” the precursors used to synthesize larger molecules, and as pathways connecting couple of singletons, possible synthetic strategies to attach a specific interesting low molecular weight singleton to another one representing, for example, a scaffold.

In our experience, the “Molecular Anatomy”’ approach allows deciphering more easily the connections between chemotypes. In particular, filtering by EF and ranking by number of connections for each cluster allow to focus the analysis on the highly connected singletons. These frameworks have high relevance, considering that they connect different chemotypes without overlapping fragments and, then, could, suggest the most significant parts of active molecules, the fragments that could be exchanged, and the bond order and the atom type relevant for SAR derivation. This approach allows to include in SAR analysis also molecules usually underestimated because singletons, or compounds with small ligand efficacy, but here connected to relevant clusters corresponding to specific series of compounds. In this way, a valuable information could be added in the SAR of this major hit series, connecting them to additional latent ones [39]. This method could be considered an extension of the already proposed compound set enrichment [17, 40, 41], based on an implementation of an higher level of abstraction, potentially able to identify new hit series connected with the conventional one.

### Case study I: SAR analysis of an HTS campaign on HDAC7

In order to better illustrate the molecular scaffold representations and the fragmentations rules that we introduced and with the intent to clarify the advantages to use the network visualization proposed for SAR evaluation, we present, as case study, the SAR analysis of the HTS campaign on HDAC7 performed for 26,092 compounds.

First, the set of nine molecular frameworks at different abstraction levels were generated for the entire dataset. For each of the nine frameworks, the EF was calculated, based on the inhibition data of the corresponding molecules; molecules were considered as active if belonging to the activity classes moderate, strong and very strong (Table 1).

**Fig. 10:**
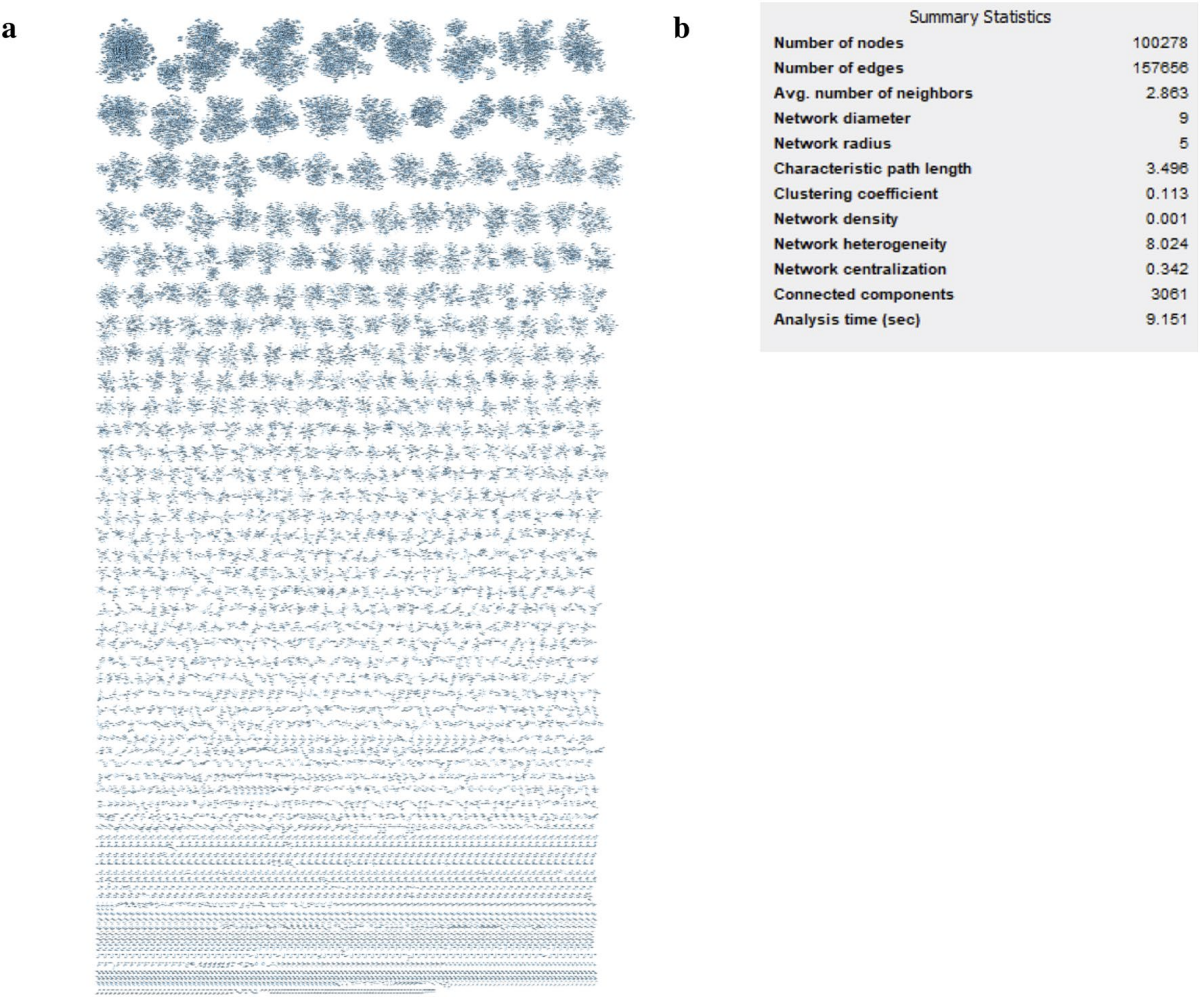
**a** Cytoscape network visualization of the dataset of 26,092 compounds with inhibition data on HDAC7; **b** main statistical parameters of the network

Figure 10 shows the complete network obtained with Cytoscape, as described in “Methods” section, for this dataset, that clearly appears a more complex case study compared to the previous one, thus chosen to show the potentiality of our approach. 3061 connected components were generated, corresponding to the clusters obtained using the most abstracted (basic wireframe) representation.

The most interesting basic wireframe in terms of SAR evaluations are selected (Additional file 1: Figure S3), filtered by the highest values of EF and number of connected active molecules, to focus the analysis on the abstracted scaffolds accounting for more actives.

**Fig. 11:**
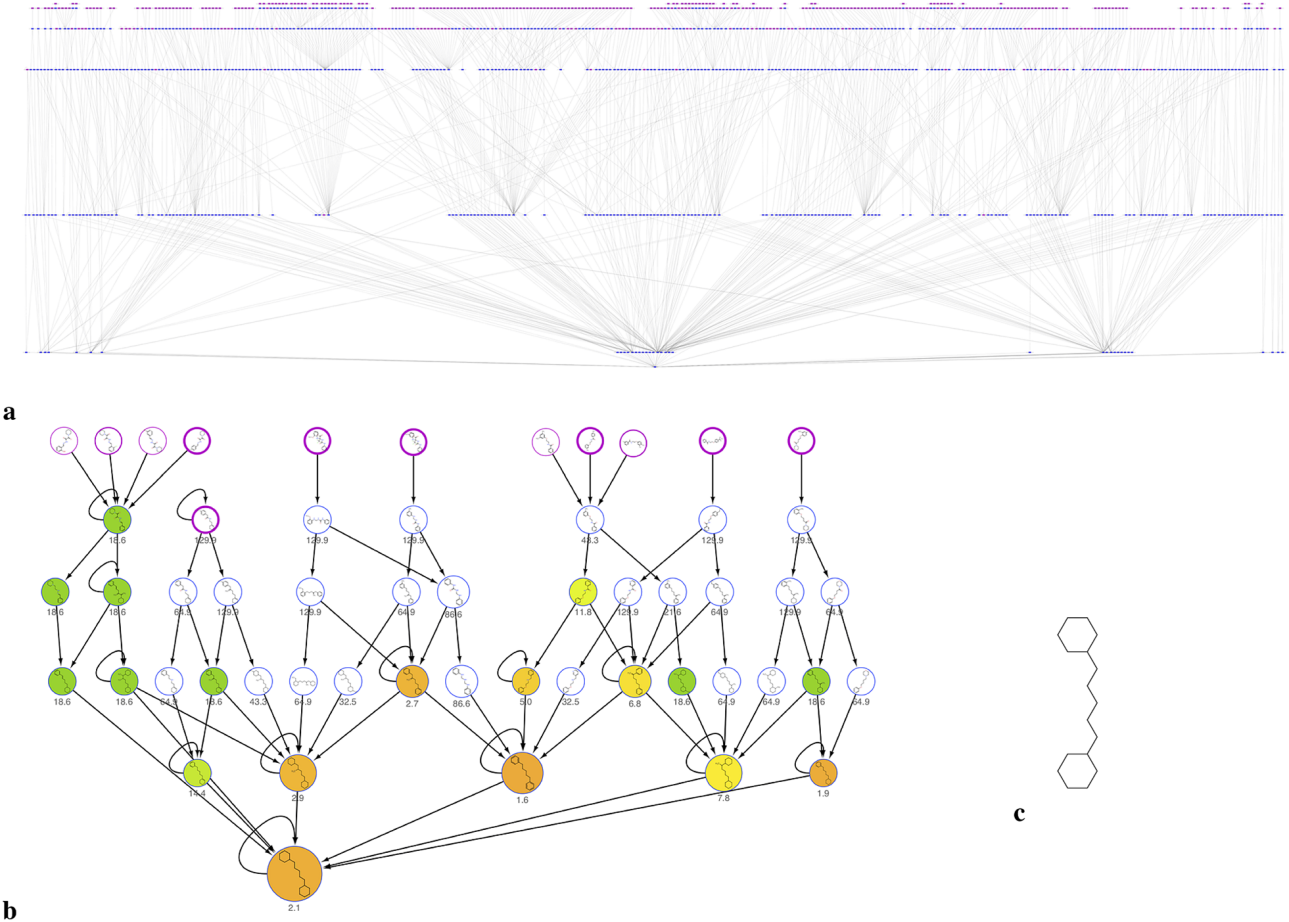
**a** Network corresponding to the cluster of molecules having the same basic wireframe shown in panel (**c**). **b** The same network is simplified by removing nodes with EF values equal to 0, that is molecules and scaffolds not relevant in terms of SAR information. Nodes correspond to the molecules, on the top of the hierarchical visualization colored in purple with a border width depending on the activity class, and to the relative molecular frameworks, colored according to the EF value reported as label of the node (from orange to green in the direction of ascending values) and sized according to the number of actives

Figure 11a reports the network corresponding to one of these selected clusters, using a hierarchical layout for a better visualization. The complexity of this specific network is due to the high number of nodes corresponding to all the molecules (on top, in light blue) and relative molecular frameworks (all other nodes) matching the basic wireframe reported in Fig. 11c. This complex network may however be considerably simplified removing nodes with EF value equal to 0, that is, removing all the nodes connected to inactive molecules. Applying this filter, a more clear and useful network can be obtained (Fig. 11b), with the most relevant dataset information. In this way, it is possible to easily extract only the interesting pathways in terms of SAR analysis, starting from a huge number of connections that ensure a complete evaluation of the structural information.

**Fig. 12:**
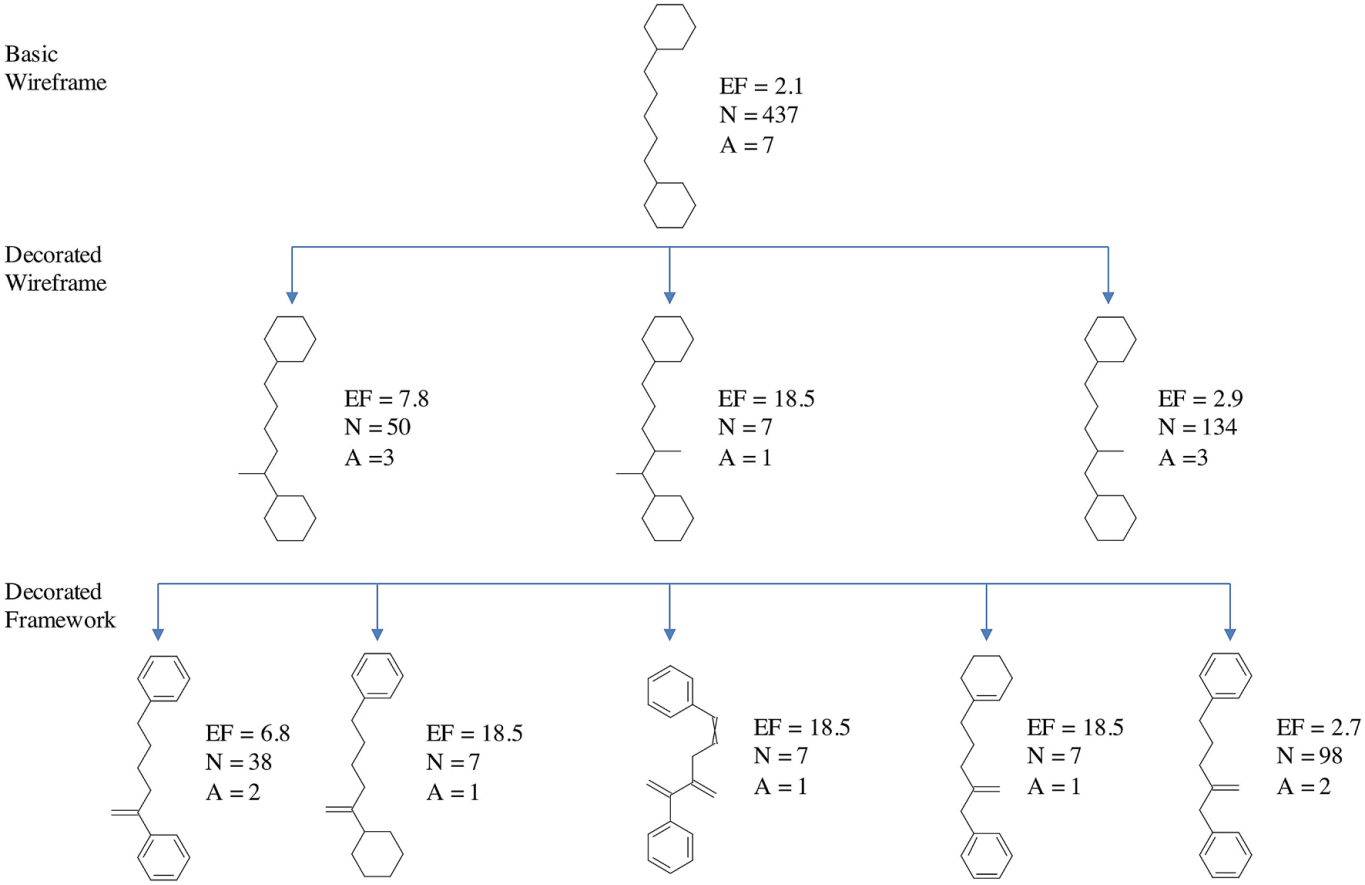
Decorated wireframes and decorated frameworks identified according to increased values of EF within the cluster analyzed in Fig. 11; this analysis allows to retrieve specific characteristics of the active compounds

In more detail, starting from the basic wireframe selected (Fig. 11c), thanks to the network visualization, more interesting sub-clusters can be identified corresponding to the decorated wireframes reported in Fig. 12. The EF values of these decorated wireframes are higher than that of the basic wireframe in common, meaning that such approach allows focusing on specific characteristics of the active compounds.

Focusing on the increase of EF, for example moving from the basic wireframe (EF = 2.1) to the first decorated wireframe (EF = 7.8), allows to highlight interesting “hotspots”, identifying a specific feature of the active molecules structure (e.g. a protruding bond in position 1 of the spacer between the two rings).

Furthermore, it is also possible to move to the less abstracted representation within the network, the decorated frameworks also reported in Fig. 12, that provide information about the bond order characteristics common to the active compounds. And so on, moving back through the network toward the lowest abstraction level is it possible to visualize the original molecules.

A first interesting consideration about these results concerns the introduction of decorations in our scaffold representations: defining a description level in which protruding bonds are added to the basic scaffold allows to better identify and distinguish the requirement essential for the activity. This point is clearly showed in Figs. 11 and 12, where moving from the basic to the decorated wireframes with higher values of EF and number of connections, it is possible to retrieve all the clusters containing the active molecules. On the other hand, 12 decorated wireframes and 37 decorated frameworks are identified in common with inactive molecules, another useful information to rationalize which scaffold decorations are responsible of decrease or even loss of activity.

Finally, we want to show how the most useful SAR information can be obtained extending the analysis and the network to the fragments. When the fragmentation rules are applied to the dataset, the network visualization of the fragmented library allows to interconnect all the molecular frameworks containing the same fragment and the EF can be recalculated for each fragment according to the activity data of all the molecules connected via the corresponding molecular frameworks.

**Fig. 13:**
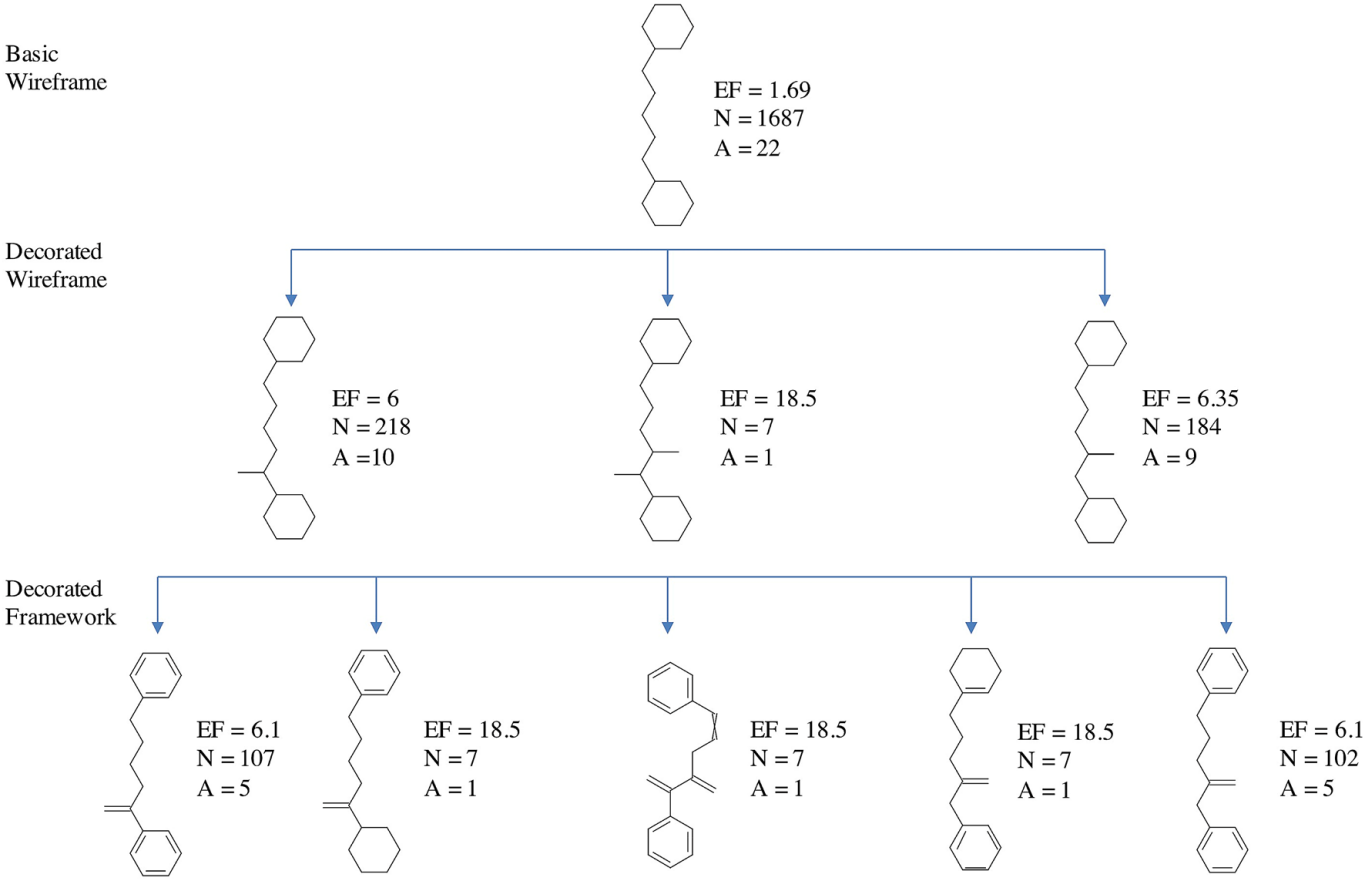
After library fragmentation, the decorated wireframes and decorated frameworks described in Fig. 12 are treated as fragments, allowing to retrieve all the molecular frameworks containing them (superframeworks). The corresponding EF values are recalculated adding the contribution of all the superframeworks

In particular, focusing the attention on the interesting structures above identified, Fig. 13 reports the same scheme of Fig. 12, with the EF values recalculated considering all the clusters identified by molecular frameworks corresponding to superscaffolds of the scaffolds visualized (superframeworks).

Comparing Fig. 12 and 13, it is possible to identify the molecular frameworks, the EF of which increases when they are considered as fragments, thus containing relevant structural characteristic of active molecules.

**Fig. 14:**
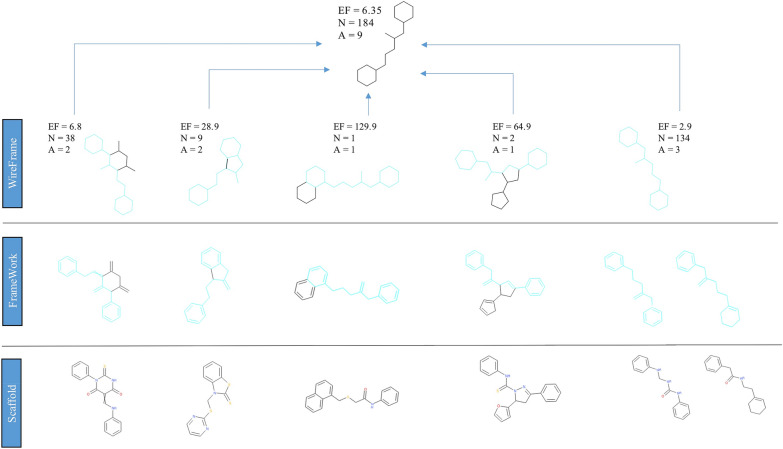
One of the decorated wireframes (colored in black, on the top) corresponds to a common fragment of five decorated wireframes (highlighted in cyan) retrieved in the fragmented library. These, in turn, are also represented into the corresponding decorated frameworks and scaffolds

To better explain the contribution of the integration of fragments and molecular frameworks in the SAR analysis, we report on top of Fig. 14, as example, the decorated wireframe of Fig. 13 showing the higher increase of EF value respect to Fig. 12 and the corresponding five decorated wireframes retrieved in the fragmented library containing it as a fragment. For each of these decorated wireframes, the EF value is reported and that of the common wireframe on top, here treated as a fragment, is recalculated, adding the contribution of the other five ones. For each decorated wireframe the corresponding decorated framework and scaffold of the active molecules are reported in the lower panel of Fig. 14.

This example shows how this approach allows to extract structural information from the different levels of scaffold's representations. The decorated wireframe on top of Fig. 14, identified on the basis of the higher EF value, represents the common pharmacophore of nine active compounds, corresponding to two rings connected by a five heavy atoms linker with an H-bond acceptor in position 2. The five decorated wireframes matching this pharmacophore enrich the information, showing that the structural flexibility can be reduced with a cyclization, involving different positions of the linker, and this information can clearly orient the design of new compounds. Moving to the level of framework, the information related to the double bonds can be added, showing that in most of the active compounds both the rings are aromatic. This information might suggest that aliphatic rings could also be included in active compounds. A simple search among the library frameworks allows to easily verify if this feature is already present in the library, in inactive compounds, otherwise it may represent a possible modification to be explored. Finally, moving to the scaffold level, the information related to the atom type can be added, showing if ketone, amide, urea or thiourea are preferred moieties in the active compounds, and thus providing useful insights for the design of new compounds.

We can conclude that, even if in this particular case study the decorated wireframe seems the most informative representation, in general the integration of all molecular frameworks and fragments in the network visualization is crucial for capturing the most relevant information in compound libraries SAR analysis.

### Performances of different molecular frameworks in terms of EF

**Fig. 15:**
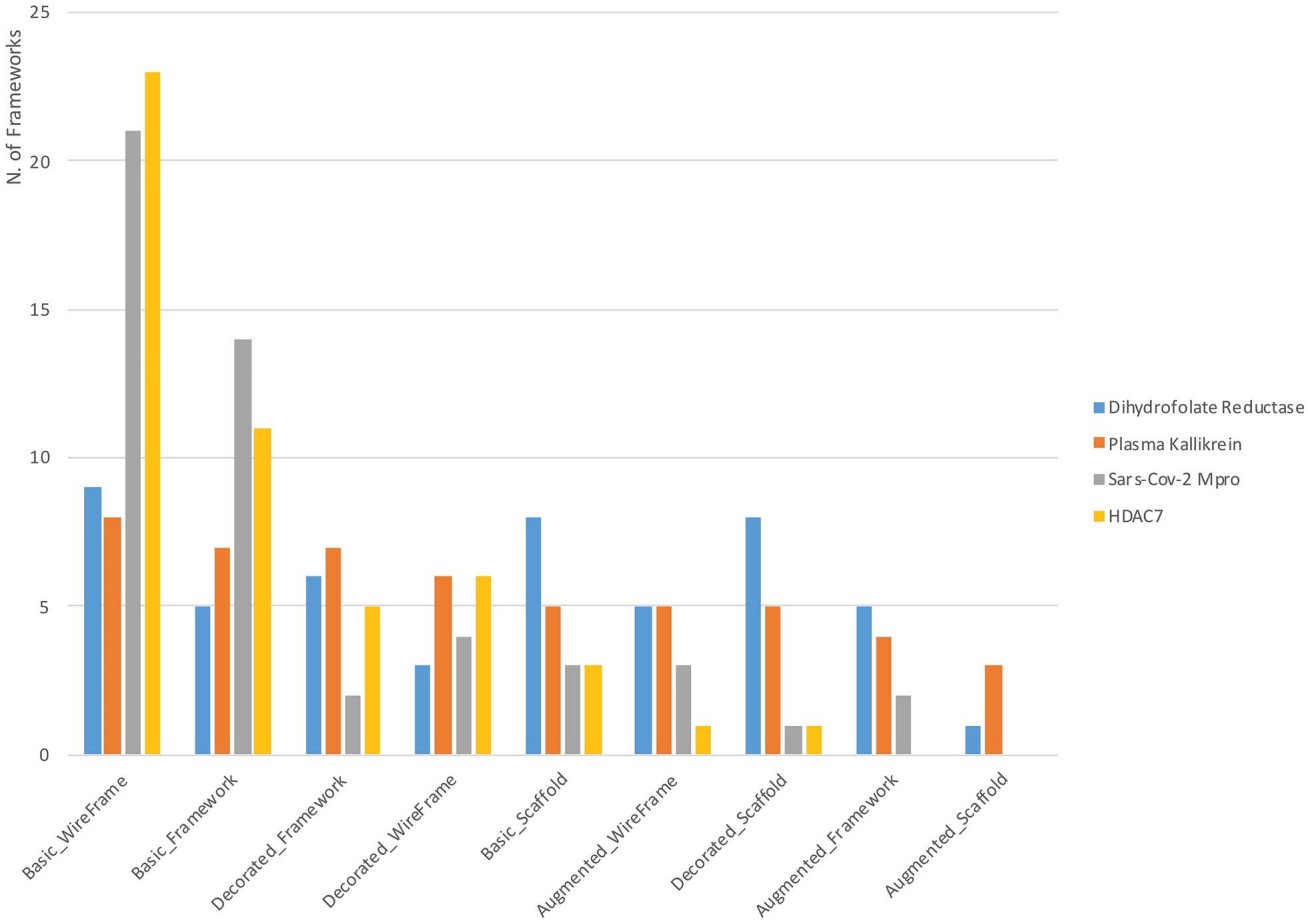
The distribution of selected frameworks with highest EF and number of connected molecules for the four datasets

In order to investigate which level of scaffold abstraction leads to the highest enrichment factors, we employed three further datasets from ChEMBL (see “Methods” section) together with the above-described HDAC7 dataset. The nine molecular frameworks were generated for all these datasets and the corresponding EF were calculated. The top 50 frameworks with the highest EF values and number of connected molecules were selected; the plot in Fig. 15 shows the number of the selected frameworks for each of the nine representations. As pointed out above, the basic wireframe often represents the molecular framework that performs better in the identification of relevant chemotypes of active molecules, in particular for larger and diverse datasets, such as Mpro and HDAC7. However, also the other representations, in some cases, are much informative, in particular for capturing the most significant features of molecules among active compound series. Therefore, the integration of the information retrieved from the different scaffold abstractions allows to more effectively map the chemical space of different types of compound datasets.

### Web interface

The “Molecular Anatomy” approach is available as a web application, freely accessible at https://ma.exscalate.eu. The user can upload a text file containing one or more compounds, encoded as canonical SMILES, to generate the molecular frameworks related to the nine molecular representations at different abstraction levels.

The output consists of four tables, resembling Additional files 2, 3, 4, 5: Table S1–S4 of this paper, named Molecular Frameworks for each SMILES, Molecular Frameworks list, Attribute file for network, Network file. Each output table can be downloaded as .csv file. Specifically, the first table contains the uploaded compounds, and the related nine molecular representations, encoded as canonical SMILES and InChIKey, listed in the same row. In the second output table, each compound and the corresponding nine molecular representations are listed as single rows. The third output table consists of the list of unique frameworks (attribute file) whereas the fourth output table (network file) lists all the parent–child relationships which can be used for the network visualization in Cytoscape, permitting an efficient navigation in the scaffold’s space that can be readily used for SAR analysis.

The dataset of 816 COX-2 inhibitors used for this study has been provided within the web interface as template file to test the application (by clicking on the “Submit Example” button).

## Conclusions

We propose “Molecular Anatomy” as a fast and flexible method for the analysis of the chemical space, library design and SAR studies.

This set of tools could be useful in the management of large compounds collections, for example in the analysis of HTS campaign results, as well as in focused libraries design. On the other side, this kind of data organization allows to efficiently analyze scaffold-activity relationship, identify relevant clusters and easily connect different chemotypes with biological activity. The limitation in the underestimation of the side chain effect can be easily circumvented combining the “Molecular Anatomy” approach with other techniques, such as matched molecular pairs (MMP) [42–44]. In this case, the identified molecular frameworks can make MMP equally or even more efficient and consistent than other methods [45, 46]. Furthermore, using MA with a higher level of abstraction, it is possible to compare effectively SAR behaviors on multiple scaffolds, or support scaffold hopping strategies.

Another interesting application, still in an early phase of evaluation, is the possibility to annotate a library according to therapeutic areas information of classified drugs, in order to accelerate the identification of target-based or disease-based libraries, using for example annotated database such as MDDR, WOMBAT [47], or public databases [48].

Furthermore, molecular frameworks can be profitably used for compounds clustering and database indexing. One of the most critical tasks in the design of large diverse libraries is the comprehensive mapping of chemical space. The generation of groups that can be considered as “series” in a medicinal chemist’s perception represents an important asset of the scaffold-based techniques. However, the clusters generated by currently available approaches generally tend to contain an elevated number of scaffolds, hampering the selection of chemical series for follow-up activities. Some improvements have been introduced to overcome this problem, for example by means of Maximum Overlapping Set (MOS) [49]. The main advantage of scaffold-based clustering techniques is that they do not require the calculation of similarity indices, nor pairwise similarities: indeed, the scaffold structure itself represents the aggregation rule, so that each molecule is assigned to a cluster regardless of the nature of the neighbors. In this sense, the approach can be defined as a “Natural Clustering” (NC) method. Another important feature is that no bias, like the average number of molecules per group or the expected number of scaffolds, has to be introduced. This is very useful when some over-represented scaffolds may drive the analysis. Therefore, MA enables the hierarchical clustering, considerably extending the potentialities of scaffold-based clustering.

Finally, NC makes relational databases ideal for chemical graph-based compound clustering applications. The composition of a specific cluster is independent from the chemical structure of the other clusters, and, once the scaffold abstraction is defined, isn’t needed to re-cluster the whole library.

Organizing molecules within a database by means of clustered SQL indices (based on the MA), can dramatically reduce the time required for substructure searches, as reported by Wilkens et al. [50] and Masciocchi et al. [51]. In our implementation, due to the higher abstraction of the frameworks and wireframes, it is also possible to further speed up substructure searches using these representations as a wild character-like query, such as “any atom” or “any bond”. Interestingly, MA are, per se, searchable molecular representations, and this allows to define local similarities in substructure searches space. For example, the scaffold of a target molecule could be searched with either a lower or higher similarity to the reference template. Besides that, it is also possible to constrain the local diversity of the scaffold by requiring, for example, the presence of a specific hydrogen bond acceptor at a given position on the scaffold, or even specifying a LogP range.

All these prospective applications make the MA approach a valuable cheminformatics tool that can considerably improve structural data analysis.

## Supplementary Information

**Additional file 1: Figure S1.** Cytoscape network visualization of the 816 COX-2 inhibitors subset where nodes includes fragments related to the basic wireframe representation, contributing to create a fully connected unique network. **Figure S2.** The fragments extracted from the basic wireframe representation, with the highest number of connections (indegree) in the Cytoscape network visualization reported in Figure S1. **Figure S3.** Selection of the most interesting basic wireframe, corresponding to the most abstracted representation in common within each cluster of the network, filtered by the highest values of EF and number of connected active molecules of the corresponding cluster.

**Additional file 2: Table S1.** Dataset of 816 COX-2 inhibitors in preclinical development or in a higher phase, described according to the nine molecular representations of “Molecular Anatomy”. Each compound and the related nine molecular representations are encoded as canonical SMILES and InChIKey, respectively, and are listed in the same row.

**Additional file 3: Table S2.** Dataset of 816 COX-2 inhibitors reported in Table S1; each compound and the related nine molecular representations are encoded as canonical SMILES and InChIKey and are listed as single rows. From this list, Tables S3 and S4 are generated.

**Additional file 4: Table S3.** List of unique frameworks obtained from the molecules in Table S2.

**Additional file 5: Table S4.** List of parent–child relationships between the nine molecular frameworks of molecules listed in Table S2.

## Data Availability

Datasets generated and analyzed during this study are included in this published article and its Additional files. The Pipeline Pilot protocol employed for the current study can be downloaded at: https://github.com/andreabeccari/Molecular_Anatomy. A reimplementation of this protocol in Knime is available at the same address. The protocol is also available as a web application, freely accessible at https://ma.exscalate.eu.

## Abbreviations

HTS: High-throughput screening
SAR: Structure activity relationships
MCS: Maximum Common Substructure
HDAC7: Histone deacetylase 7
MF: Molecular frameworks
EF: Enrichment factor
MOS: Maximum Overlapping Set
NC: Natural Clustering
MMP: Matched molecular pairs
MOS: Maximum Overlapping Set
