# Supplementary material for: “Molecular Anatomy”: a new multi-dimensional hierarchical scaffold analysis tool
Source: J Cheminform. 2021 Jul 23;13:54. doi: 10.1186/s13321-021-00526-y (PMC8299179; doi:10.1186/s13321-021-00526-y)

**Additional file 1**

Molecular anatomy:
A New Multi-Dimensional Hierarchical Scaffold Analysis tool

Candida Manelfi^a^, Marica Gemei^a^, Carmine Talarico^a^, Carmen Cerchia^b^, Anna Fava^a^, Filippo Lunghini, and Andrea Rosario Beccari^a*^

^a^ Dompé Farmaceutici SpA, L'Aquila, Via Campo di Pile, 67100, Italy

^b^ Department of Pharmacy, University of Naples “Federico II”, 80131 Napoli, Italy

* To whom correspondence should be sent.

Email: andrea.beccari@dompe.com

**Figure S1**: Cytoscape network visualization of the 816 COX-2 inhibitors subset where nodes include fragments related to the basic wireframe representation, contributing to create a fully connected unique network.


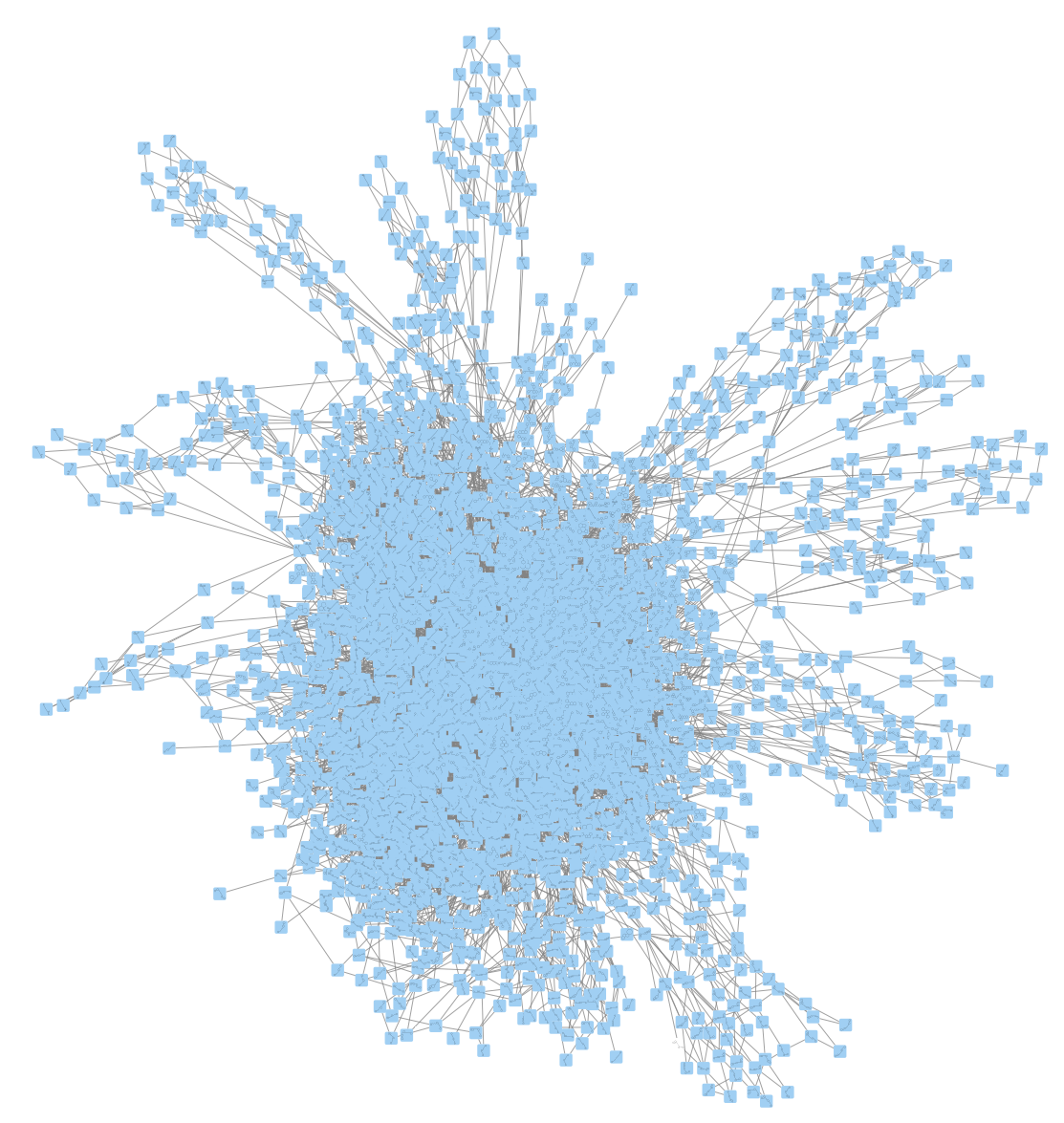


**Figure S2.** The three fragments, extracted from the basic wireframe representation, with the highest number of connections (indegree) in the Cytoscape network visualization reported in Figure S1.


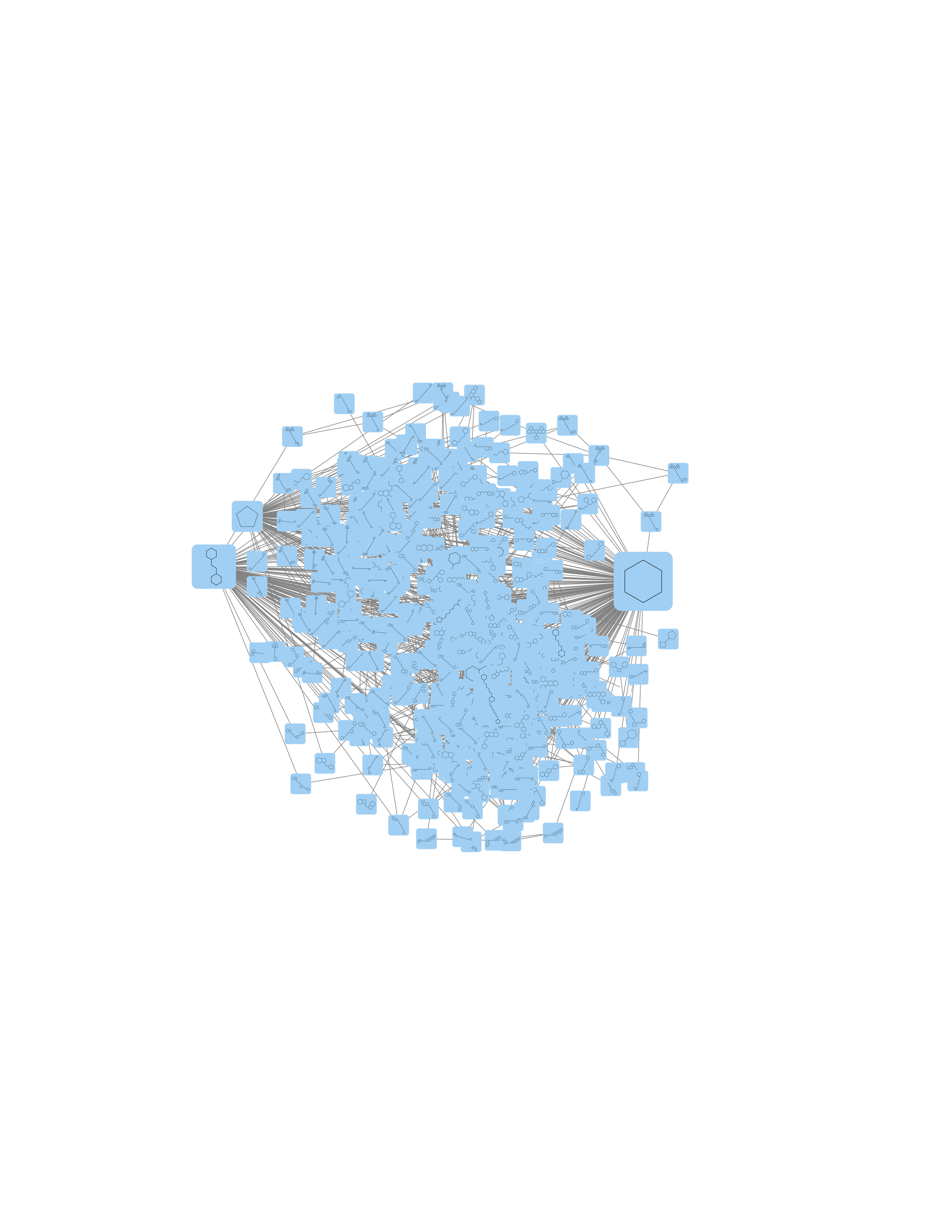


**Figure S3**: Selection of the most interesting basic wireframe, corresponding to the most abstracted representation in common within each cluster of the network, filtered by the highest values of EF and number of connected active molecules of the corresponding cluster.


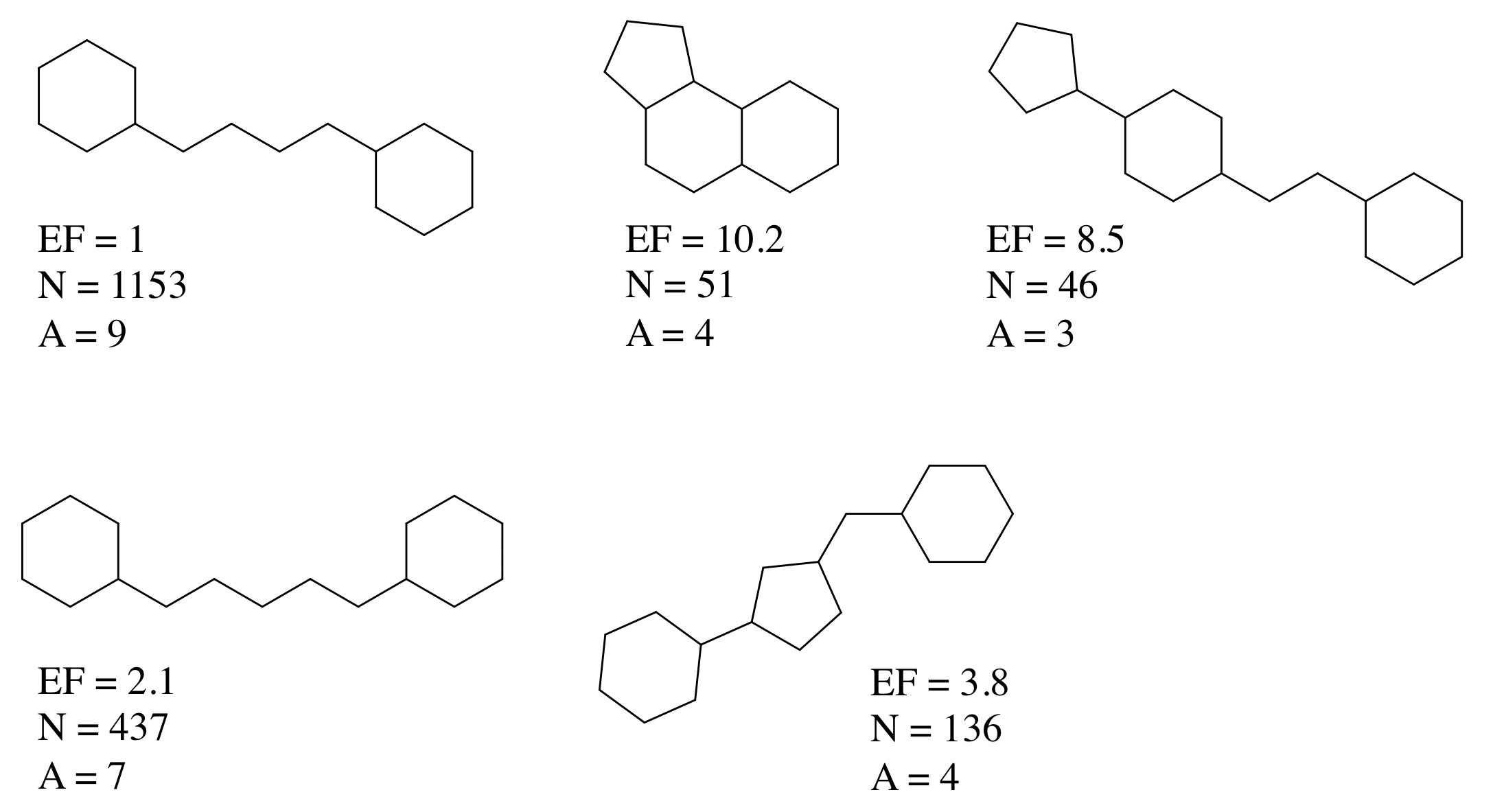

Supplement: Supplementary file 1 — Additional file 1: Figure S1. Cytoscape network visualization of the 816 COX-2 inhibitors subset where nodes includes fragments related to the basic wireframe representation, contributing to create a fully connected unique network. Figure S2. The fragments extracted from the basic wireframe representation, with the highest number of connections (indegree) in the Cytoscape network visualization reported in Figure S1. Figure S3. Selection of the most interesting basic wireframe, corresponding to the most abstracted representation in common within each cluster of the network, filtered by the highest values of EF and number of connected active molecules of the corresponding cluster. [file 13321_2021_526_MOESM1_ESM.docx]
